# Prevalence and Molecular Characterization of Methicillin-Resistant *Staphylococcus aureus* ST398 Resistant to Tetracycline at a Spanish Hospital over 12 Years

**DOI:** 10.1371/journal.pone.0072828

**Published:** 2013-09-05

**Authors:** Mariana Camoez, Josep M. Sierra, Miquel Pujol, Ana Hornero, Rogélio Martin, M. Angeles Domínguez

**Affiliations:** 1 Department of Microbiology, Hospital Universitari de Bellvitge, Barcelona, Spain; 2 Department of Infectious Diseases, Hospital Universitari de Bellvitge, Barcelona, Spain; 3 Epidemiology of Bacterial Infections Group, IDIBELL, Barcelona, Spain; 4 Department of Pathology and Experimental Therapeutics, Universitat de Barcelona, Barcelona, Spain; 5 Spanish Network for the Research in Infectious Diseases (REIPI RD06/0008), Instituto de Salud Carlos III, Madrid, Spain; Universitätsklinikum Hamburg-Eppendorf, Germany

## Abstract

Methicillin-resistant *Staphylococcus aureus* (MRSA) ST398, associated with livestock animals, was described in 2003 as a new lineage infecting or colonizing humans. We evaluated the prevalence and molecular characteristics of MRSA ST398 isolated in the Hospital Universitari de Bellvitge from January 2000 to June 2011. Tetracycline resistant (Tet-R) MRSA isolates from single patients (pts) were screened by *Sma*I-pulsed field gel electrophoresis (PFGE). Nontypable MRSA strains by *Sma*I (NT*_Sma_*_I_)-MRSA were further analysed by *Apa*I-PFGE, *spa*, SCC*mec, agr*, MLST typing, and by DNA microarray hybridization. Among 164 pts harboring Tet-R MRSA, NT*_Sma_*_I_-MRSA ST398-*agr*I was found in 33 pts (20%). Although the first pt was detected in 2003, 22/33 pts (67%) were registered in the 2010–2011 period. Ten pts (30%) were infected and cancer was the most frequent underlying disease. In one case, death was due to MRSA-ST398-related infection. Five pulsotypes (A–E) were detected using *Apa*I-PFGE, with type A accounting for 76% of the strains. The majority of the studied isolates presented *spa* type t011 (70%) and SCC*mec* type V (88%). One strain was *spa* negative both by PCR and microarray analysis. Forty-nine percent of the studied isolates showed resistance to 3 or more antibiotic classes, in addition to beta-lactams. Ciprofloxacin resistance was 67%. Tet-R was mediated by *tet*(M) and *tet*(K) in 26 isolates. All isolates lacked Panton-Valentine Leukocidin production, as well as other significant toxins. This study displays the molecular features of MRSA-ST398 clone and shows the increase in tetracycline resistance together with arise in MRSA-ST398 isolates infecting or colonizing patients in our clinical setting.

## Introduction

Methicillin-resistant *Staphylococcus aureus* (MRSA) is an important human pathogen causing nosocomial and community-acquired infections worldwide [1]. In recent years, community acquired MRSA strains that are genetically unrelated to the traditional hospital have emerged [2]. MRSA Clonal Complex (CC) 398 associated with livestock (LA-MRSA) has been described as a new clonal lineage infecting or colonizing humans in several countries around the world [3].

According to several studies, human's exposure to livestock constitutes a risk-factor for carriage of MRSA CC398 strains an' development of a possible infection [4]. Carriage prevalence in livestock farming profession is very high [1], [5], but some strains have been detected in people without risk factors [6]. The majority of the MRSA sequence type (ST) 398 strains are related to skin and soft tissue infections (SSTIs) [7]. However severe infections can occur and have been reported in Europe, Asia, and the United States [8], [9], [10], [11]. Although there is little information on the percentage of this clone associated to human infections, the Netherlands, Belgium, Denmark, Germany and Austria appear to be the countries that have more cases [8]. MRSA-ST398 was first reported in Spain in 2010 associated to a skin lesion [12].

Some microbiologic traits define isolates belonging to the MRSA-ST398 clone: chromosomal DNA cannot be restricted by *Sma*I enzyme and CC398 strains generally present resistance to tetracycline, which is commonly used in pig farming [1], [13], [14]. The absence of certain important virulence factors such as Panton-Valentine Leukocidin (PVL) and Toxic Shock Syndrome Toxin (TSST) seems to be common in CC398 isolate. Nevertheless, this clone has been associated with both animal and human disease [14].

The aims of this study were to evaluate the prevalence and molecular characteristics of MRSA ST398, isolated in the Hospital Universitari de Bellvitge from January 2000 to June 2011.

## Materials and Methods

### Clinical Setting

The present study has been approved by the Clinical Research Ethics Committee of the Hospital Universitari de Bellvitge. The Ethics Committee granted an exemption on obtaining the patients informed consent as this retrospective study focused on bacteria characterization and did not require any specific patient involvement. The study was conducted at the Hospital Universitari de Bellvitge (HUB), a 900-bed tertiary care academic institution located in the Barcelona metropolitan area, Spain. It is a reference centre for adult patients with approximately 35,000 admissions per year providing medical and surgical care for a population of 1,000,000 inhabitants. Most of the patients live and work in urban areas; however, the HUB is also the reference hospital for some rural population living to the south of the city of Barcelona. All episodes of infection or colonization by MRSA of ST398 were reviewed. Clinical information including patient age, sex, associated diseases and infection source were recorded.

### Bacterial Strains

*S. aureus* isolates were identified using standard tests: catalase, latex agglutination (Microgen Staph, Microgen Biproducts, Camberley, England) and tube coagulase test (Staph-ase, bioMérieux, Marcy l-Étoile, France).

The average rate of total MRSA among *S. aureus* clinical isolates in our hospital was 21% for the 2000–2011 period (ranging from 15% in 2001 to 26% in 2006). A total of 184 MRSA tetracycline resistant (Tet-R) strains were isolated from single patients from January 2000 to December 2011, accounting for 6% of all MRSA recovered during this time period. One hundred and sixty-four of these Tet-R MRSA strains were available for this study. We failed to re-culture the remaining 20 isolates so they could not be included in the study.

### Susceptibility Testing

Susceptibility testing was carried out by the disk-diffusion method following the Clinical and Laboratory Standards Institute (CLSI) recommendations [14], [15]. The antibiotics tested were: penicillin (10 units), oxacillin (1 µg), cefoxitin (30 µg), erythromycin (15 μg), clindamycin (2 μg), gentamicin (10 μg), tobramycin (10 μg), rifampicin (5 μg), tetracycline (30 μg), trimethoprim-sulfamethoxazole (1.25/23.75 μg), chloramphenicol (30 μg), ciprofloxacin (5 μg), teicoplanin (30 μg), quinupristin/dalfopristin (15 μg), and linezolid (30 μg). Vancomycin and daptomycin susceptibility was studied by broth microdilution.

### Molecular Typing and DNA microarray hybridization

#### Pulsed Field Gel Electrophoresis (PFGE)

The Tet-R MRSA isolates available for the study (n = 164) were tested for *Sma*I restriction as previously described [16]. Nontypable MRSA strains by *Sma*I (NT*_Sma_*_I_)-MRSA were further re-analyzed by *Apa*I-PFGE with pulse times from 0.5 to 15 s for 20 h. The resulting restriction patterns by *Apa*I enzyme, were interpreted both by visual inspection, using the criteria of van Belkum *et al.* [17], and by analysis with the FINGERPRINTING TM II software, version 3.0 (BioRad Laboratories, Inc., Madrid, Spain). The dendogram was generated using the unweighted-pair group with arithmetic averages method based on Dice coefficients, where optimization and band position tolerance were both set at 0.7%. PFGE type clusters were defined by a similarity coefficient of 80%.

#### Multilocus sequence typing (MLST)

MLST was performed on representative strains of each *Apa*I-PFGE subtype as described by Enright *et al* [18]. *S. aureus* MLST database (http://www.mlst.net) was used to assign sequence types.

#### agr typing

The detection of *agr* polymorphism was performed in all strains using the multiplex PCR as described previously [19].

#### SCCmec typing

The staphylococcal cassette chromosome *mec* (SCC*mec*) type was characterized for all the NT*_Sma_*_I_-MRSA isolates, using the multiplex PCR strategy developed by Milheiriço *et al*. [20].

#### spa typing

All NT*_Sma_*_I_-MRSA strains were characterized by *spa* typing as described previously [21] using the Ridom StaphType software, version 1.4 (Ridom GmbH Münster, Germany) and the *spa* types were assigned according to the Ridom web server (http://www.spaserver.ridom.de). The BURP algorithm was used to calculate *spa* clonal complexes (*spa*CC) with the defaults parameters set by the software, i.e. *spa* types shorter than five repeats were excluded and *spa* types were clustered in the same *spa*CC if cost was less or equal to six.

#### DNA microarray hybridization

DNA microarray hybridization (Staphy-Type Kit, Alere Technologies Ltd, Jena, Germany; stripe version) was conducted in all NT*_Sma_*_I_-MRSA strains following the manufacturer's guidelines. The protocols and procedures have been previously described in detail [22]. Briefly, this microarray contains 334 probes, including approximately 180 different genes and their allelic variants. Target genes, primers and probes were previously published in the Electronic supplementary material of Monecke *et al.* [23].

## Results

**Figure 1:**
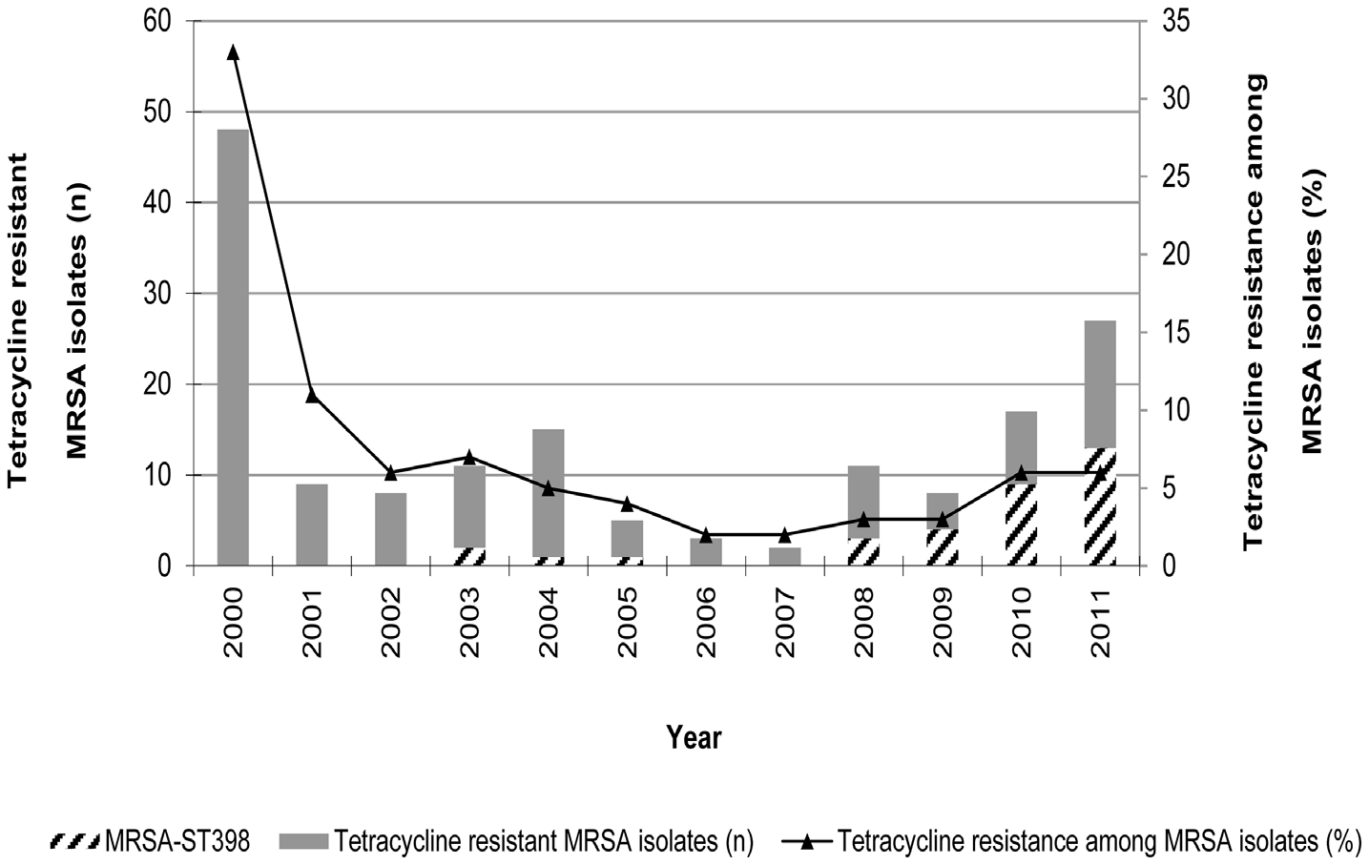
Distribution of 164 tetracycline resistant MRSA isolates studied from 2000 to 2011 in Hospital Universitari de Bellvitge, Barcelona, Spain.

Among 164 Tet-R MRSA isolated between 2000 and 2011 from single patients, 33 (20%) NT*_Sma_*_I_-MRSA strains were found. All 33 isolates belonged to ST398 and showed an *agr* type I. The first MRSA-ST398 isolate was identified in 2003. Yearly distribution of MRSA-ST398 isolates is shown in Figure 1. It is noted that out of 33 MRSA-ST398, 22 (67%) were isolated during the 2010–2011 period.

**Figure 2:**
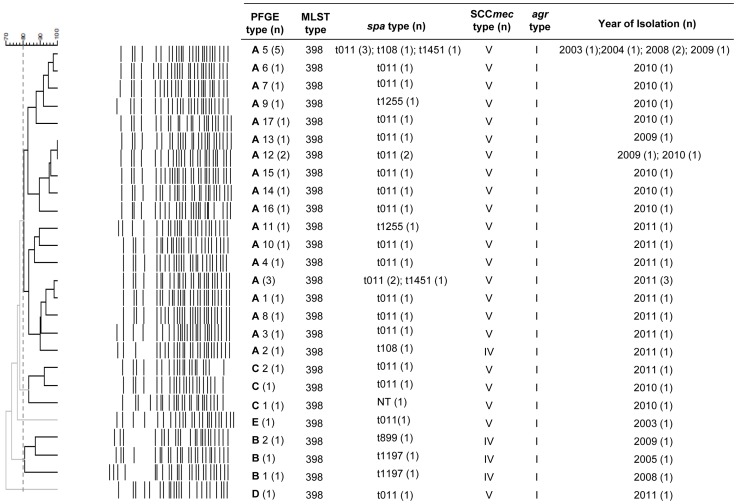
Cluster analysis of Pulsed-field Gel Electrophoresis (PFGE) *ApaI* macrorestriction fragments of methicillin-resistant *Staphylococcus aureus* ST398 isolates followed by multilocus sequence typing (MLST), staphylococcal protein A (*spa*), staphylococcal cassette chromosome (SCC*mec*), accessory gene regulator (*agr*) typing and year of isolation. For dendogram construction, optimization and band position tolerance were both set at 0.7%. The cut-off value to define the PFGE patterns was set at 80% similarity.

Digestions with *ApaI* restriction of MRSA-ST398 isolates provided five unrelated pulsotypes (A–E) using a cut-off at 80% similarity (Figure 2). Among these five major clusters, type A accounted for 76% (25/33) of the studied strains (Figure S1). SCC*mec* type V was carried by 29 (88%) isolates and SCC*mec* type IV was only found in four isolates. A total of six different *spa* types were detected, with t011 as the dominant type present in 23 (70%) isolates. The other *spa* types identified were: t1255 (n = 2), t1197 (n = 2), t108 (n = 2), t1451 (n = 2) and t899 (n = 1). One strain was *spa* negative both by PCR and microarray analysis (Figure 2). The BURP algorithm assigned all spa types, except singleton t899, to *Spa*-CC011 (n = 31; 94%).

The percentage of antibiotic resistance among the 33 isolates of ST398 was as follows: resistance to erythromycin was found in 11 isolates (33%), to clindamycin in 16 (48%), to tobramycin in 8 (24%) and to ciprofloxacin in 22 (67%). No resistance was found to vancomycin, daptomycin, rifampicin or mupirocin. Fourteen antibiotic resistance patterns were found among the MRSA-ST398 isolates (Table 1). The most frequent combination of resistances were: tetracycline plus ciprofloxacin (8/33; 24%) and tetracycline, ciprofloxacin, erythromycin plus clindamycin (6/33; 18%), with 49% (16/33) of the isolates being resistant to three or more antibiotic groups, in addition to beta-lactams.

**Table 1:** Antibiotic resistance patterns and resistance genes of the 33 ST398-MRSA isolates recovered in Hospital Universitari de Bellvitge, 2000–2011.

Resistance pattern^1^ (n)	Tetracycline resistance genes		Other resistance genes					No. Isolates
	*tet*(K)	*tet*(M)	*erm*(A)	*erm*(C)	*aacA-aphD*	*aadD*	*cat*	
TET, CIP (8)	*+*	*+*	*−*	*−*	*−*	*−*	*−*	6
	*−*	*+*	−	*−*	*−*	*−*	*−*	2
TET (7)	*+*	*+*	−	*−*	*−*	*−*	*−*	3
	*−*	*+*	−	*−*	*−*	*−*	*−*	3
	*+*	*−*	−	*−*	*−*	*−*	*−*	1
ERY, CLI, TET, CIP (6)	*+*	*+*	*−*	*+*	*−*	*−*	*−*	6
CLI, TET, CIP (2)	*+*	*+*	*−*	*−*	*−*	*−*	*−*	2
CLI, TOB, TET, CIP (1)	*+*	*+*	*−*	*−*	*−*	*+*	*−*	1
ERY, CLI, GEN, TOB, TET, CIP (1)	*+*	*+*	*+*	*+*	*+*	*−*	*−*	1
ERY, CLI, TOB, TET, CIP(1)	*+*	*+*	*−*	*−*	*−*	*+*	*−*	1
CLI, GEN, TOB, TET, CIP, SYN (1)	*+*	*+*	*−*	*−*	*+*	*+*	*−*	1
ERY, CLI, CLO, TOB, TET (1)	*+*	*+*	*−*	*+*	*−*	*+*	*+*	1
ERY, CLI, TOB, TET (1)	*+*	*+*	*−*	*+*	*−*	*+*	*−*	1
ERY, CLI, TOB, TET, SXT, CIP (1)	*+*	*+*	*−*	*+*	*−*	*+*	*−*	1
TOB, TET, CIP (1)	*−*	*+*	*−*	*−*	*−*	*+*	*−*	1
CLI, TET (1)	*+*	*+*	*−*	*−*	*−*	*−*	*−*	1
CLO, TET (1)	*+*	*+*	*−*	*−*	*−*	*−*	*+*	1
**Total No of Isolates**								**33**

1 TET, tetracycline; CIP, ciprofloxacin; CLI, clindamycin; TOB, tobramycin; GEN, gentamicin; SYN, synercid; ERY, erythromycin; CLO, chloramphenicol; SXT, sulfamethoxazole-trimethoprim.

Antibiotic resistance genes, as determined by DNA hybridization, are shown in Table 1. All 33 strains harboured *mec*A and the beta-lactamase gene *bla*Z. Tetracycline resistance in most of the cases (26/33) was mediated by both *tet(*K) and *tet(*M) genes. In addition to tetracycline resistance, erythromycin and clindamycin combined resistance was seen in 10 (30%) isolates carrying the gene *erm*(C)– one of these isolates harbored *erm*(A) in addition to *erm*(C)-, and one isolate was negative for these genes. Other macrolide and lincosamide resistance genes reported to occur in staphylococci such as *erm*(B), *msr*(A), *mph*(C), *vga*(A) and *lnu*(A) were negative in this subgroup of strains. Five isolates (15%) showed an unusual erythromycin-susceptibility/clindamycin-resistance pattern. No genes associated with this resistance phenotype such as *vga*(A) or *lnu*(A) were identified by microarray in the MRSA-ST398 strains studied. Two isolates showing resistance to gentamicin harbored the gene *aacA/aphD*. Tobramycin resistance was mediated by *aacA/aphD* (1/8) and by *aadD* (7/8) genes. Chloramphenicol resistance was detected in 2 strains carrying the *cat* gene.

Regarding the presence of genes coding for virulence factors, none of the MRSA-ST398 isolates harbored the genes encoding PVL (*luKS-PV*/ *lukF-PV*), enterotoxins (*sea* to *ser*), exfoliative toxins (*etA*/*etB*/*etD*), *egc* cluster (*seg/sei/sem/sen/seo/seu*) or TSST (*tst*). A single isolate carried the *seb* gene encoding enterotoxin B. The vast majority of the strains were positive for haemolysins genes *hla*, *hlb, hld*, *hlgA*, *hlgB* and *hlgC*. All but one, were positive for the *cna* gene responsible by the collagen-binding adhesion. Genes carried on mobile genetic elements and involved in immune evasion such as *scn* (staphylococcal complement inhibitor), *sak* (staphylokinase) and *chp* (chemotaxis inhibitory protein) were identified in a single isolate of *spa* type t899. Fibronectin-binding protein A gene (*fbnA*) was detected in 9/33 (27%) of the isolates. All isolates carried genes codifying capsule type 5.

The clinical characteristics of patients infected or colonized by MRSA-ST398 are shown in Table 2. In total, there were 27 men (82%) and 6 women (18%) with a mean age of 65 years (range 41 to 92). In 23 patients (70%), the isolation of MRSA-ST398 was considered colonization, more frequently as result of active surveillance for nasal MRSA colonization (17/23; 78%). MRSA-ST398 was causing infection in 10 patients. Eight patients experienced non-invasive infections: in four cases the source of the infection was the respiratory tract and in the other four cases the patients experienced non-invasive skin and soft tissue (SST) infections. Two patients, with prior community-acquired MRSA-ST398 nasal colonization, went through invasive infections: one of them suffered a catheter-related bacteremia and the other a subdural empyema following a surgical drainage of a subdural haemorrhage. The patient with the catheter-related bacteremia was the sole case where death could be related to the MRSA-ST398 infection. The most frequent underlying disease was cancer, with this condition found in 11 patients, of whom 6 were colonized and 5 were infected by MRSA-ST398.

**Table 2:** Clinical features of 33 patients colonized or infected by ST398-MRSA.

	No. (%) or mean ± SD with variable	
**Men**	27 (81)	
**Age, years (range)**	65 (41–92)±12.9	
**Rural area**	24 (77)	
**Underlying conditions**		
Cancer	11 (33)	
**Sample Source**	**Colonization**	**Infection**
Blood	0 (0)	1 (3)
Respiratory tract	1 (3)	4 (12)
Skin and Wound	4 (12)	4 (12)
Nares	18 (55)	0 (0)
Central Nervous System	0 (0)	1 (3)
**Total number (%)**	23 (70)	10 (30)

## Discussion

Since 2005, several studies have been carried out focusing on livestock-associated methicillin-resistant *Staphylococcus aureus* (LA-MRSA) isolates, mainly on isolates of CC398 [1], [3]–[5], [10], [13], [24], [25]. Human colonization with LA-MRSA was first described among swine farmers in France and The Netherlands in 2003 [4], [9]. This is the first retrospective study of MRSA-ST398 in Spain covering a period from 2000 to 2011. In order to evaluate the prevalence and molecular characteristics of this clone in our hospital, one hundred and sixty-four isolates obtained between 2000 and 2011 from different sources were investigated. Prior to year 2000 Tet-R was very high among the MRSA isolated from the patients admitted to the HUB. This was due to the presence of an endemic clone – the Iberian clone (ST247) that was resistant to several antibiotics, including tetracycline. In the following years, tetracycline resistance dropped, often below 5%. Antibiotic susceptibility analysis revealed an unexpected diversity of resistance profiles among MRSA-ST398 isolates. In total, 14 different resistance patterns were seen with 10 of them represented by only one or two isolates. Of note, 49% of the isolates exhibited resistance to three or more antimicrobial agents, in addition to methicillin-resistance. Ciprofloxacin resistance was high (67%) considering the low percentage of resistance to this antibiotic exhibited by other MRSA community clones. Spanish [7] and Belgian [5] studies described resistance to fluoroquinolones of 58% and 83% respectively. However, in other European countries [14], USA [10] or Canadian studies [26], the resistance percentage to fluoroquinolones is very low or even inexistent. The veterinary use of quinolones in Spain is difficult to assess. The public data available on the website of the Spanish Ministry of Health (*Ministerio de Sanidad, Politica Social y Igualdad*) (www.aemps.gob.es) only describes the total amount of antimicrobials sold for veterinary purposes, and does not distinguish between the therapeutic use of antibiotics and the use of antibiotics to enhance growth. In any case, total sales of quinolones was about 50 tons in 2009, much lower than the sales of other antibiotics such as tetracyclines (350 tons), sulfonamides (250 tons) or beta-lactams (180 tons) during the same year.

The combination of *tet*(K) and *tet*(M) resistance genes was commonly seen among the 33 MRSA-ST398 isolates. The methylase *erm*(C) gene was present in 10 out of 11 erythromycin resistant strains. Resistance to cotrimoxazole was found in a single isolate, whereas in other MRSA-ST398 studies cotrimoxazole resistance was more prevalent and in some cases exceeded 80% [5], [27].

Some strains showed the unusual erythromycin-susceptibility/clindamycin-resistance pattern. This phenotype seems to be related to animal clonal lineages of *S. aureus* animals and has been associated with the presence of plasmid-borne resistance genes *vga*(A), *vga*(C), *lnu*(A) or *lnu*(B) [28], [29]. Some resistance genes described in MRSA CC398 strains such as *erm*(T), *vga*(B), *vga*(C), *lnu*(B), *dfrSI, dfrK, dfrG* and *tet*(L) [27] were not included in the DNA microarray applied in this study.

The dominant *spa* type t011 in our collection has been commonly found in other studies, both in Spain [7], and in other countries [8], [25], [30], [31]. There was a single isolate of *spa* type t899, with only two repeats in common with t011. Therefore, this t899 isolate did not cluster in *Spa*-CC011 after BURP analysis. As described previously [30], this could be explained by the acquisition of a large DNA region which includes the *spa* gene, from a remote *S. aureus* clone. *spa* type t899 has been described in association to ST9, a clone also described in samples of animal origin [32]. The linkage of the same *spa* type in two different ST types would suggest exchange of genetic material between two clones of animal origin.

The presence of genes coding for virulence factors was very poor in our collection. None of the studied isolates carried the PVL – encoding genes *lukF-PV* and *lukS-PV*, contrasting to studies from Sweden and China that report PVL-positive isolates in patients who had no previous contact with animals [6], [11]. Generally, MRSA-ST398 lacks certain important virulence factors for humans [13]. In our study genes involved in immune evasion (*scn*, *sak a*nd *chp*) were only detected in the single t899 isolate. These virulence factors are active only against the innate immune system in humans [33]. The lack of these virulence factors may partially explain why these strains do not appear to be highly infectious for humans, and usually are associated with SST infections [25]. However, a few severe infections by ST398 have been sporadically published in several countries, such as pneumonia or bacteraemia [24], [31]. Even though the majority of the patients in our study were colonized by MRSA-ST398 (70%), four SST infections and four respiratory tract infections were detected in our series. In addition, two invasive infections, bacteraemia and subdural empyema, were also detected, in patients showing previous nasal colonization. Death was related to the MRSA-ST398 infection in the bacteremic patient: an 84-year-old woman who was hospitalized in the HUB because of a thoracic aortic aneurysm. One month after the surgery, the patient experienced a febrile episode and MRSA-ST398 was recovered from blood and from the central venous catheter tip. The isolate was resistant to tetracycline and beta-lactams. One week after treatment with vancomycin, the patient died. The isolates causing infection did not differ from the colonizing isolates, regarding genotype, virulence or antibiotic resistance profile.

In our study, some data could not be collected properly such as the contact with animals, a risk factor for infection by LA-MRSA. However, a high percentage (73%) of the patients lived in or near to a rural environment. Another limitation was the selection of the isolates to be studied by the presence of tetracycline resistance. Although this is a common feature among MRSA-ST398 [7], this approach could underestimate the number of isolates belonging to CC-398 in HUB.

In conclusion, in the last two years (2010–11) the number of MRSA-ST398 isolates infecting or colonizing patients increased significantly in our setting, as well as the increase in tetracycline resistance. The emergence of this clonal lineage has also been reported in other countries in Europe [8], [24], [25], [34]. These studies showed a remarkable increase in the proportion of LA-MRSA isolates, including outpatients and primary health care patients, which were not covered in our hospital-based study. According to our results, the majority of studied isolates carried the genes encoding haemolysins and adhesion cellular factors, but other virulence factors usually found among *S. aureus* were not detected. Phenotypic expression of antibiotic resistance was variable among the MRSA-ST398 isolates and nearly half of the isolates were resistant to multiple antibiotics. In addition, patients harbouring this clone were often debilitated by underlying diseases, such as cancer. Due to the increased public health interest about MRSA-ST398, further studies should be conducted to record risk factors from infected or colonized patients by this lineage such as routes of transmission and association with animals.

## Supporting Information

Figure S1 **Pulsed-field Gel Electrophoresis (PFGE) of** ***ApaI*** **macrorestriction fragments of methicillin-resistant** ***Staphylococcus aureus*** **ST398 isolates showing a PFGE pattern A5 followed by multilocus sequence typing (MLST), staphylococcal protein A (** ***spa*** **), staphylococcal cassette chromosome (SCC** ***mec*** **), accessory gene regulator (** ***agr*** **) typing and year of isolation data.** For dendogram construction, optimization and band position tolerance were both set at 0.7%. The cut-off value for designing genotypes was set at 80%.
(TIF)
