# Supplementary figures and images for: Prevalence and Molecular Characterization of Methicillin-Resistant Staphylococcus aureus ST398 Resistant to Tetracycline at a Spanish Hospital over 12 Years
Source: PLoS One. 2013 Sep 5;8(9):e72828. doi: 10.1371/journal.pone.0072828 (PMC3764106; doi:10.1371/journal.pone.0072828)

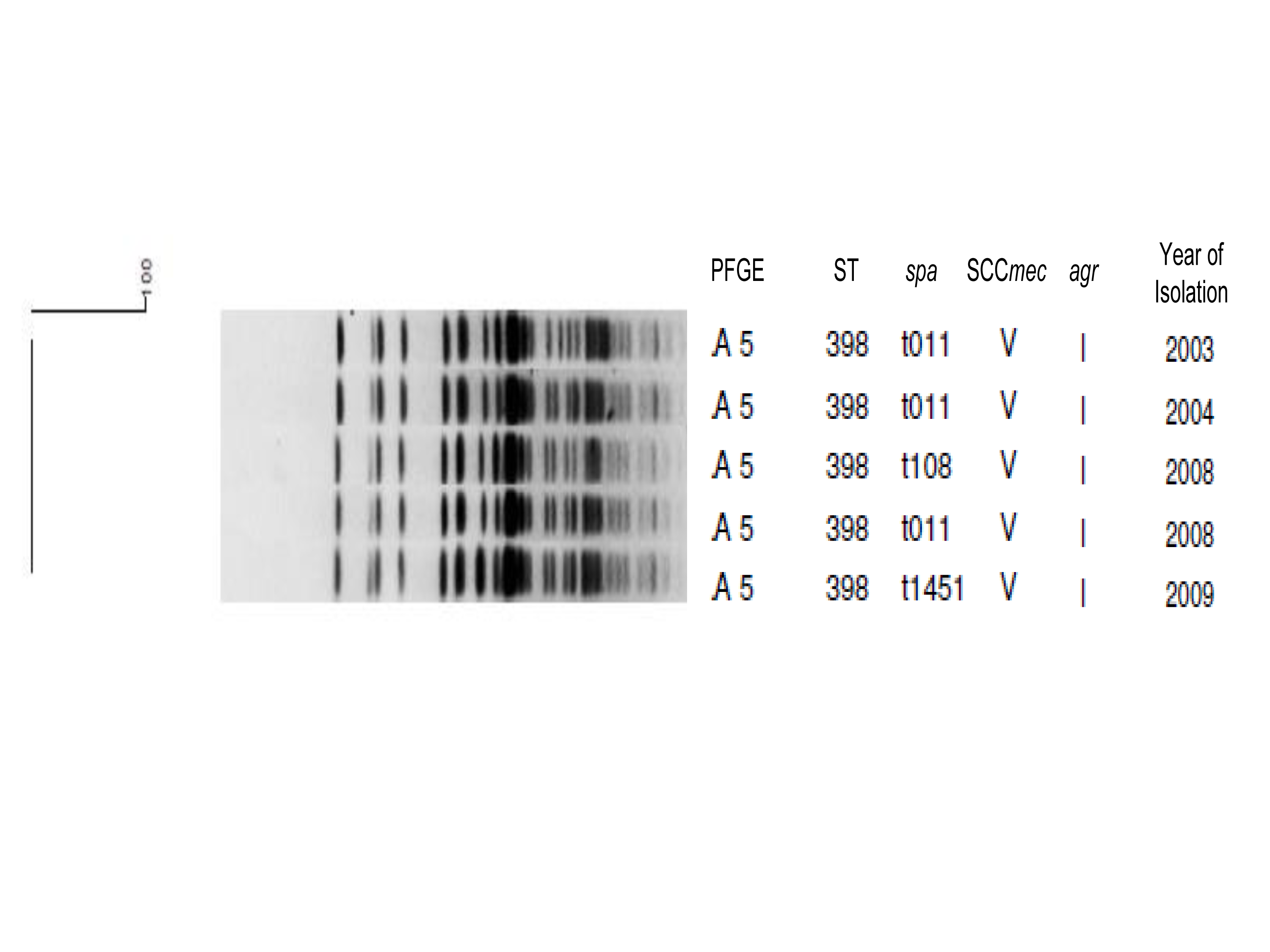

Supplement: Figure S1 — Pulsed-field Gel Electrophoresis (PFGE) of ApaI macrorestriction fragments of methicillin-resistant Staphylococcus aureus ST398 isolates showing a PFGE pattern A5 followed by multilocus sequence typing (MLST), staphylococcal protein A ( spa ), staphylococcal cassette chromosome (SCC mec ), accessory gene regulator ( agr ) typing and year of isolation data. For dendogram construction, optimization and band position tolerance were both set at 0.7%. The cut-off value for designing genotypes was set at 80%. (TIF) [file pone.0072828.s001.tif]
